# The Samaritan Free Hospital

**Published:** 1902-04-26

**Authors:** 


### THE SAMARITAN FREE HOSPITAL.

From 5 o'clock till 20 minutes past on Tuesday, April 15th, it was doubtful whether there would be a quorum for the annual general meeting, and the secretary cast many an anxious glance from the Board-room window in the direction of the front door.

"There is only one way," he remarked, "of ensuring a good attendance, and that is to put something of a controversial nature on the agenda." But there was nothing controversial to put. Several things are badly wanted: for instance, the out-patients' department is inadequate; there is not enough room in the operating theatre for the visiting doctors to watch operations without getting in the way of the surgeon and nurses; and, lastly, the nurses need more accommodation. But towards all these objects there is only £10 in hand (the secretary said he had never known such a dull time), and, moreover, the expiration of a lease of certain cottages at the back must be awaited, so a plain invitation had to be sent, with quite a harmless agenda. So each of the 250 London and suburban governors whose attendance would have been "esteemed a favour" trusted that the other 249 would accept the invitation; hence the anxiety of the secretary and the deadly dulness of the meeting.

"One more," he said joyfully, as there was a ring at the bell, and at nearly half-past five there were six gentlemen in the room, three medical representatives were collected from the wards, and the meeting began. Dr. C. H. F. Routh took the chair. The report was taken as read, and was adopted. The committee were re-elected *en bloc*, without any protest from Mr. J. R. Diggle, M.A., J.P., who thought a year ago that only those who had attended more than three committee meetings should stand. But perhaps the attendances were better in 1901. Mr. Diggle's amendment this year was carried unanimously; he proposed, on behalf of the Committee of Management, that they should be empowered to add to their own committee and to the house committee during the year if vacancies arose. All the gentlemen present were members of the Committee of Management.

The past year has been a difficult one, subscriptions having been £37 less than in 1900, and donations £1,640 less. Consequently the year, beginning with a balance of £110, ended with a deficit of £391. The legacies were only £545, instead of £1,376 as in 1900. But while money shrinks, work increases; there were 486 new in-patients instead of 471, as in 1900, and of out-patients there were 7,227. These came chiefly from London, while the in-patients were from all parts of the country. In accordance with the special wish of the Hospital Sunday Fund, an inquiry officer watches against abuses of the charity; special inquiries were made in 135 cases, and eight were rejected.

At this hospital the acting as well as the consulting staff are represented on the Management Committee, owing to an alteration made in 1900. Dr. W. R. Rogers, after nearly 40 years' active and consulting service, has passed away; Dr. Amand Routh has retired from the post of senior physician after 21 years, and has joined the consulting staff, of which his father, Dr. C. H. F. Routh, is also a member.

The smooth working of the daily routine, the report says is largely owing to the hearty co-operation of the medical and surgical staff, the matron, and the nursing and household staff. Many kind friends have sent gifts, and the lady visitors have as usual been most attentive and kind to the patients.

